# Foxc Transcription Factors Directly Regulate Dll4 and Hey2 Expression by Interacting with the VEGF-Notch Signaling Pathways in Endothelial Cells

**DOI:** 10.1371/journal.pone.0002401

**Published:** 2008-06-11

**Authors:** Hisaki Hayashi, Tsutomu Kume

**Affiliations:** Division of Cardiovascular Medicine, Vanderbilt University Medical Center, Nashville, Tennessee, United States of America; Ecole Normale Superieure, France

## Abstract

**Background:**

Recent studies have shown that in the developing embryo, arterial and venous identity is established by genetic mechanisms before circulation begins. Vascular endothelial growth factor (VEGF) signaling and its downstream Notch pathway play critical roles in arterial cell fate determination. We have recently shown that Foxc1 and Foxc2, two closely related Fox transcription factors, are essential for arterial cell specification during development by directly inducing the transcription of Delta-like 4 (Dll4), a ligand for Notch receptors. However, the basic mechanisms whereby the VEGF and Notch signaling pathways control transcriptional regulation of arterial-specific genes have yet to be elucidated.

**Methodologies/Principal Findings:**

In the current study, we examined whether and how Foxc transcription factors are involved in VEGF and Notch signaling in induction of Dll4 as well as the Notch target gene Hey2 in endothelial cells. We found that Foxc1 and Foxc2 directly activate the Hey2 promoter via Foxc binding elements. Significantly, Foxc2 physically and functionally interacts with a Notch transcriptional activation complex containing Su(H) and Notch intracellular domain to induce Hey2 promoter activity. Moreover, activation of the Dll4 and Hey2 promoters is induced by VEGF in conjunction with either Foxc1 or Foxc2 more than by either component alone. VEGF-activated PI3K and ERK intracellular pathways modulate the transcriptional activity of Foxc proteins in Dll4 and Hey2 induction.

**Conclusions/Significance:**

Our new findings demonstrate that Foxc transcriptional factors interact with VEGF and Notch signaling to regulate arterial gene expression in multiple steps of the VEGF-Dll4-Notch-Hey2 signaling pathway.

## Introduction

Advances in understanding the basic mechanisms of the formation and remodeling of blood vessels have led to the identification of several signaling molecules that are critical for vascular development. In particular, conclusive evidence demonstrates that during embryonic development, the arterial-venous identity of endothelial cells lining the blood vessels is established by genetic mechanisms prior to the onset of circulation [1]–[3]. In this process, vascular endothelial growth factor (VEGF) signaling activates the Delta-Notch pathway, including Notch1 and its ligand, Delta like 4 (Dll4), in arterial endothelial progenitors. Notch signaling subsequently upregulates the expression of Hey transcription factors, thereby specifying an arterial cell fate. Whereas mutant mice for Hey2 (also known as CHF1, Herp1, Hrt2, Hesr2 or gridlock in zebrafish) show no obvious vascular abnormalities with multiple cardiac defects [4]–[6], compound Hey1; Hey2 mutant embryos have impaired arterial-venous specification [7], [8], similar to those seen in mutant mice for Notch receptors and ligands, including Dll4 [9]–[11]. In contrast to arterial cell specification, the nuclear orphan receptor COUP-TFII, which is expressed in a subset of venous endothelial precursors, induces a venous cell fate by suppressing the expression of arterial-specific genes [12].

We have recently shown that Foxc1 and Foxc2, two closely related Fox transcription factors [13]–[16], are essential for vascular development [17]–[19]. Compound *Foxc1; Foxc2* mutants have defective vascular remodeling of primitive blood vessels and abnormal vascular connections between arteries and veins, arteriovenous malformations [17], [18]. It should be noted that such arteriovenous malformations similarly develop in endothelial cells of mutant mice and zebrafish in which Notch signaling is defective [8], [9], [11], [20], [21]. Indeed, endothelial cells of compound *Foxc1; Foxc2* homozygotes fail to induce arterial-specific genes, including *Dll4* and *Hey2,* whereas venous markers such as COUP-TFII and EphB4 are normally expressed in these mutants [18]. These results suggest the lack of Foxc1 and Foxc2 results in a failure of arterial cell specification during development. On the other hand, overexpression of Foxc1 and Foxc2 upregulates the expression of arterial-specific genes in cultured endothelial cells. Most importantly, Foxc proteins directly activate the Dll4 promoter through a Fox-binding element (FBE) that is conserved between human and mouse. Taken together, Foxc transcription factors act upstream of Notch signaling in arterial specification [18]. However, it remains unknown whether VEGF signaling directly interacts with Foxc-mediated transcriptional control in arterial gene expression. Moreover, as we have recently demonstrated that Foxc1 and Foxc2 induce expression of the chemokine receptor CXCR4 [13], Foxc proteins are likely to regulate multiple arterial-specific genes, including Hey2.

The aim of the present study is to define the molecular mechanisms by which Foxc transcription factors control arterial gene expression. We show here that Foxc1 and Foxc2 directly activate the Hey2 promoter via FBEs in endothelial cells. Remarkably, Foxc2 binds to a Notch transcriptional mediator, Suppressor of Hairless [Su(H)], and directly contributes to Notch-dependent activation of the Hey2 promoter. Furthermore, we demonstrate that VEGF-mediated PI3K and ERK/MAPK pathways modulate the activity of Foxc proteins in the activation of the Dll4 and Hey2 promoters. Taken together, our new findings provide the mechanistic basis for transcriptional regulation of the arterial program in endothelial cells.

## Materials and Methods

### Reagents

Wortmannin was purchased from Invitrogen. LY294002, PD98059 and U0126 were purchased from Promega. Mouse VEGF_164_ was purchased from R & D Systems.

### Isolation of Pulmonary microvascular endothelial cells

Foxc2 mutant mice [17] and endothelial-specific Foxc1 mutant mice [13] were described previously. Pulmonary microvascular endothelial cells (PMVECs) were isolated from adult lungs of Foxc2+/− and endothelial-specific Foxc1 mutant mice, as described previously [22].

### Real-time RT-PCR

Isolation of total RNA from PMVECs of endothelial-specific Foxc1 mutant mice and Foxc2+/- mice and cDNA synthesis were performed using the RNeasy Mini Kit (Qiagen) and iScript (Bio-Rad). Real-time PCR were carried out using the SYBR GREEN PCR Master Mix (ABI) and i-Cycler (Bio-Rad) according to the manufacturers' instructions. Each data was normalized by the expression level of peptidylprolyl isomerase A (Ppia), an internal control. PCR primers used are Hey2-sense: 5′-CCTGTCTCCCAGGCTACACT-3′, Hey2-antisense: 5′-GGCAGTGGTAGCTATTCTCCTG-3′, Ppia-sense: 5′-CAAATGCTGGACCAAACACA-3′, and Ppia-antisense: 5′-TGCCATCCAGCCATTCAGTC-3′. Results are reported as mean+s.d. of triplicate experiments from 3 samples per each group. P values were determined by Student's t-test (*p<0.05).

### Construction of expression vectors

The expression vectors for Foxc1, Foxc2, caFoxc1, caFoxc2 and Smad3 were described previously [23]. An expression vector for NICD, pCS2+mN1 IC(V1744)wt, was provided by Dr. Kopan (Washington University, St. Louis). An insert for GST-NICD was amplified by PCR using primers (sense: 5′-CGGGATTCGTGCTGCTGCCCGCAAGCGCCGG-3′, antisense: 5′-GGAATTCTTATTTAAATGCCTCTGGAATGTGGG-3′; BamHI and EcoRI sites underlined, respectively) and pCS2+mN1 IC(V1744)wt as a template DNA. An insert for GST-Su(H) was amplified by PCR using primers (sense: 5′-CGGGATCCATGGCGCCTGTTGTGACAGG-3′, antisense: 5′-GGAATTCTTAGGACACCACGGTTGCTG-3′; BamHI and EcoRI sites underlined, respectively) and the Su(H) expression vector (Invitrogen; Full-Length Mammalian Gene Collection) as a template DNA. The PCR fragments for GST-NICD and GST-Su(H) were then subcloned into the BamHI and EcoRI sites of pGEX2T (GE Healthcare) using T4 DNA ligase (New England Biolab), followed by sequence confirmation.

### Construction of luciferase reporters

The Hey2 promoter (nucleotides –6.8 kb to the Nru site +10) was isolated from a mouse genomic DNA bacterial artificial chromosome clone (BACPAC Resource Center at Children's Hospital Oakland Research Institute) and inserted into the SmaI site of pBluescriptKS+ vector (Invitrogen). The fragment after confirming its sequence was subsequently inserted into pGL3-basic reporter (Promega), resulting in FULL-LUC reporter. A series of deletion constructs for NdeI (−5.8 kb), SacI (−2.0 kb) and NheI (−0.5 kb) were generated by digesting FULL-LUC with these restriction enzymes, respectively. Mutant luciferase reporters, FxMT-LUC (from ACCAATAGAAAGCCACAC to ACCGGGAGAGGGCCACAC), NotMT-LUC (from CGTGGGAAA to CGTGTTCCA) and FNMT-LUC (in combination with FxMT and NotMT), were generated using the NheI-LUC reporter as a template DNA and PfuTurbo DNA polymerase (Stratagene) according to the instructions of QuickChange XL Site-Directed Mutagenesis Kit (Stratagene). The mutated fragments were enzymatically digested, purified, and subcloned into linearized pGL3-basic vector at the NheI and NruI sites, followed by sequence confirmation. The Dll4 luciferase reporter was described previously [18].

### Cell culture, transfection, and reporter assay

Immortalized mouse embryonic endothelial cells (MEECs) [24] and primary bovine aortic endothelial cells (BAECs) were cultured in Dulbecco's modified Eagle's medium supplemented with 10% fetal bovine serum. Transfection of plasmid DNA was performed using Lipofectamine and Plus reagent for BAECs or Lipofectoamine 2000 (Gibco-BRL) for MEECs according to the manufacturer's instructions. For luciferase reporter assay, pRL-CMV reporter plasmid (Promega) containing the *Renilla* luciferase gene as an internal control was co-transfected with the firefly luciferase reporter constructs. All transfections were carried out in triplicate in gelatin coated 24-well plates. For VEGF treatment, the transfected cells were cultured in the presence of 0.1 % serum (serum-starved) for 1 h before treatment with VEGF alone or VEGF and chemical compounds at 24 h after transfection and harvested after additional 24 h. Luciferase assays were carried out using the Dual-Luciferase assay kit (Promega). Data are expressed as means+s.d. of three independent experiments in triplicates.

### Chromatin Immunoprecipitation (ChIP) assay

Cultured MEECs were washed with PBS and treated with formaldehyde to cross-link protein to DNA. Cellular lysates were obtained by scraping, followed by pulse ultrasonication to shear cellular DNA. After centrifugation, supernatants containing sheared chromatin were incubated with anti-Foxc1 or Foxc2 antibody (Abcam) overnight, followed by addition of Protein A+G Sepharose (Santa Cruz). After elution, immune complexes were subsequently treated with proteinase K at 55°C for 1.5 h and extracted with phenol/chloroform and chloroform. Immunoprecipitated DNA was analyzed by PCR using specific primers corresponding to the Hey2 promoter (sense: 5′-GTCCGCCCCTCCATATAAC-3′ and antisense: 5′-CTACTGTCGCCTAGCGGAAC-3′) or the Dll4 promoter (sense: 5′-GGCAAAAACTCCAAGTACGC-3′ and antisense: 5′-CACCTGCCGGTCAATAAATC-3′).

### GST pull-down assay

GST-fusion proteins were produced in *Escherichia coli* and purified by glutathione-conjugated agarose beads (Sigma-Aldrich). Proteins for Foxc1, Foxc2 and NICD were generated by in vitro transcription and translation using the TNT-coupled reticulocyte lysate system (Promega) and [^35^S]methionine. Different combinations of GST-fusion proteins and ^35^S-labeled proteins were mixed (1:1) with lysis buffer (50mM Tris, pH8.0, 150 mM NaCI, 0.1% SDS, 0.5% and sodium deoxycholate with Protease Inhibitor Cocktail tablets) for 1 h at 4°C. Beads were centrifuged, washed with lysis buffer, boiled with SDS-sample buffer, and subjected to SDS-PAGE. Expression levels of GST-fusion proteins were determined by Coomassie Brilliant Blue (CBB) staining. Autoradiography was subsequently processed using PharosFX Molecular Imager System (Bio-Rad) to detect specific binding of ^35^S-labeled proteins to GST-fusion proteins.

## Results

### Foxc transcription factors activate the Hey2 promoter in endothelial cells

**Figure 1:**
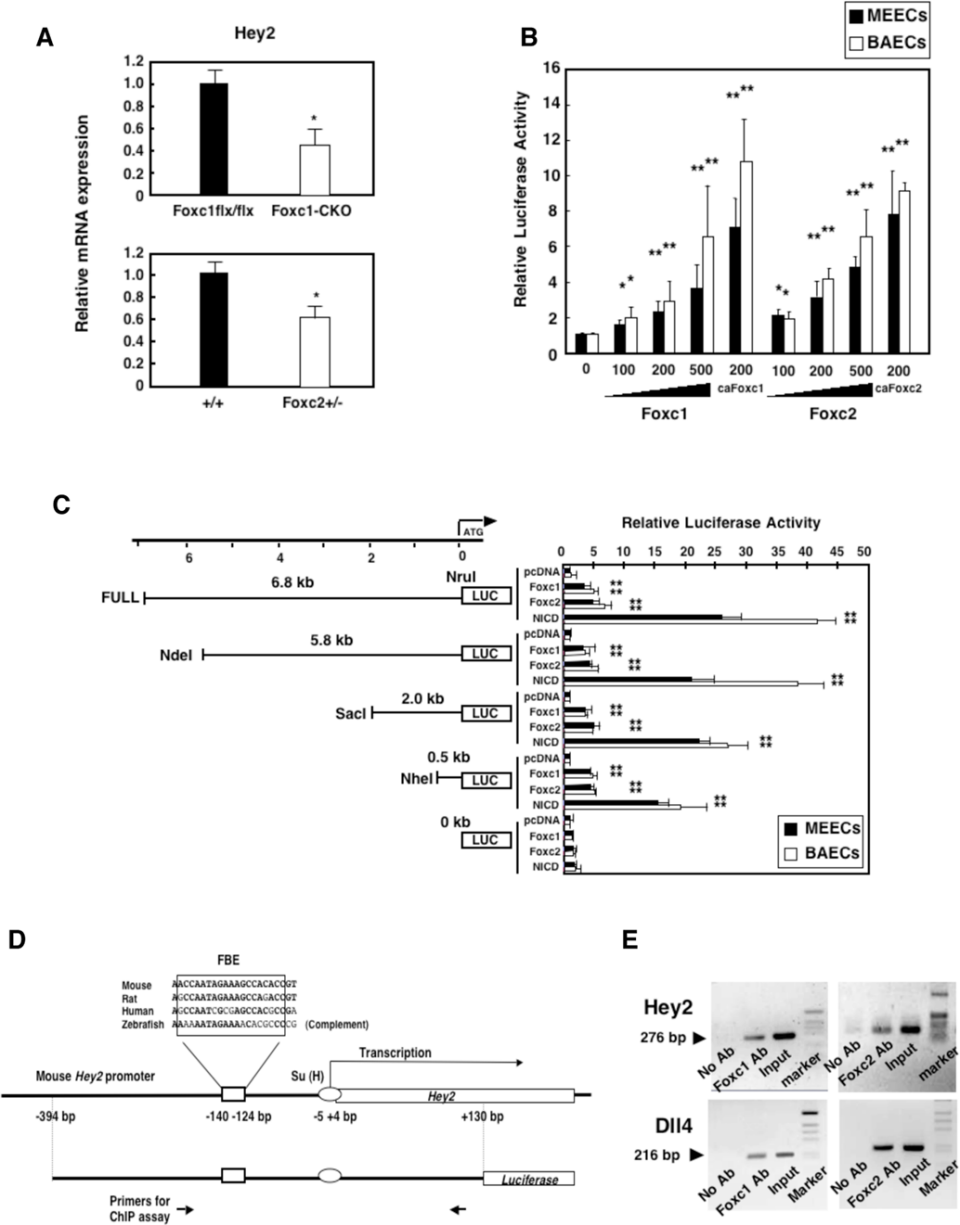
Foxc1 and Foxc2 directly regulate Hey2 expression. (A) Reduced expression of Hey2 in Foxc-mutant endothelial cells. Total RNA was prepared from PMVECs isolated from control (Foxc1flx/flx) and endothelial-specific Foxc1 conditional mutant (Foxc1-CKO) mice (upper panel) as well as wild-type and Foxc2+/− mice (lower panel). Relative mRNA levels of Hey2 were measured by real-time RT-PCR. Values are mean+s.d. of triplicate experiments from 3 samples per each group. Statistical significance was determined by Student's t-test (*p<0.05 versus control). (B) MEECs and BAECs were transfected with Hey2 luciferase reporter (6.8 kb; FULL-LUC) along with different amounts (0–500 ng) of Foxc expression vectors. Constitutively active forms of Foxc1 and Foxc2 (caFoxc1 and caFoxc2, respectively) were also transfected as positive controls. Values are means+s.d. of 3 experiments in triplicates. Statistical significance was determined by Student's *t*-tests (*p<0.05, **p<0.01 versus control). (C) A series of Hey2-luciferase constructs was transfected with or without Foxc expression vectors. NICD was also transfected as a control. Note that the minimum promoter (0.5 kb) of Hey2 still retained Foxc- and NICD-induced promoter activity. (C) Tandem FBEs conserved among several species in the minimum Hey2 promoter. Conserved nucleotides are shown in bold face. The previously reported Su(H) site [25] is located adjacent to the FBEs. (E) Foxc1 and Foxc2 bind to the endogenous promoters of Hey2 and Dll4 in endothelial cells. MEECs were subjected to ChIP assays with PBS (No Ab) or anti-Foxc antibodies, and immunoprecipitated DNA was analyzed by PCR using specific primers for the Hey2 promoter shown in (C) or the Dll4 promoter [18].

We have recently demonstrated that Hey2 expression is downregulated in compound Foxc1−/−; Foxc2−/− mutant embryos, whereas Hey2 transcription is significantly induced by overexpression of Foxc genes in endothelial cells [18]. In fact, we found that mRNA levels of Hey2 were significantly reduced in PMVECs isolated from adult lungs of endothelial-specific Foxc1 mutant mice [13] as well as Foxc2+/− mice [17], compared with the control PMVECs (Figure 1A). Despite transcriptional activation of mouse Hey2 mediated via canonical Notch signaling, ie, through the interaction of Notch1 intercellular domain (NICD) and [Su(H)] [25], the identification of additional transcription factors involved in Hey2 expression remains to be elucidated. Therefore, we first tested whether Foxc1 and Foxc2 could directly regulate Hey2 promoter activity. Using a 5′-upstream region (6.8 kb) of the Hey2 gene, we found that Foxc1 and Foxc2 dose-dependently activated the Hey2 promoter in two endothelial cell lines, immortalized MEECs and primary BAECs (Figure 1B). Consistently, constitutively active forms of Foxc1 and Foxc2 (caFoxc1 and caFoxc2, respectively) [23], which include the N-terminal activation domain and the DNA binding domain, further activated the Hey2 promoter (Figure 1B). Analysis of a series of deletion constructs of the Hey2 promoter revealed that Foxc-mediated activity of the shortest promoter (0.5 kb) remained almost intact compared with the longer promoter regions (Figure 1C), suggesting that this region is responsible for the induction. In addition, NICD strongly activated the shortest Hey2 promoter, as previously shown [25]. Based on consensus Fox binding sequences that overlap each other, RYMAAYA [26], [27] and WAARYAAAYW [28] (R = A or G; Y = C or T; M = A or C; W = A or T), the Hey2 promoter element (0.5 kb) contains two FBEs that are located adjacent to the previously reported binding site for Su(H) [25]: ACCAATA (or CCAATAGAAA) and GAAAGCC (or AAAGCCACACC) (Figure 1D). Although these FBEs do not completely match the consensus sequences, Fox proteins can bind to sequences partially matched to the consensus sequences [26]. In accordance with these results, specific binding of Foxc1 and Foxc2 proteins to the endogenous Hey2 promoter containing the FBEs was detected in MEECs by ChIP assays, as in the case of the reported FBE in the Dll4 promoter [18] that matches the consensus RYMAAYA sequence (Figure 1E). Together, these data suggest that Hey2 is a novel downstream target of Foxc1 and Foxc2.

### Foxc2 interacts with the Notch pathway to regulate Hey2 promoter activity

**Figure 2:**
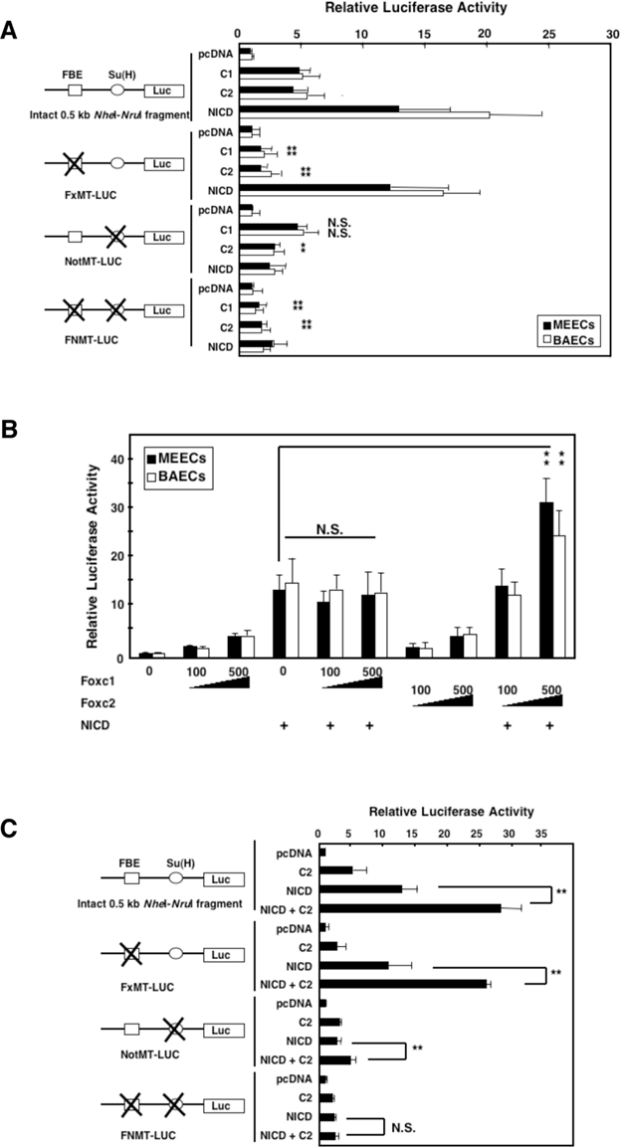
Foxc2 and NICD synergistically activate the Hey2 promoter. (A–C) Luciferase assays. Values are means+s.d. of 3 experiments in triplicates. Statistical significance was determined by Student's *t*-tests. (A) Foxc-mediated promoter activity was analyzed by Hey2 luciferase constructs containing mutations in the FBEs, the proximal Su(H) sites, or both. *p<0.05, **p<0.01 versus the intact promoter with either Foxc1 or Foxc2. N.S., non-significant. (B) Synergistic activation of the Hey2 promoter by combinations of Foxc2 and NICD. **p<0.01 versus NICD. N.S., non-significant. (C) Foxc2 and NICD synergistically induce Hey2 expression largely via the Su(H) site and partially via the FBE. **p<0.01 versus NICD. N.S., non-significant.

It has previously been shown that the proximal Su(H) site located in the shortest Hey2 promoter (Figure 1D) is critical for Notch-mediated Hey2 induction [25]. Therefore, we examined the effects of Foxc on the FBEs and Su(H) in the Hey2 promoter in endothelial cells (Figure 2A). In both MEECs and BAECs, Hey2 promoter activity mediated by Foxc1 and Foxc2 was significantly attenuated by mutating the FBEs (FxMT-LUC), indicating that Foxc proteins directly regulate the Hey2 promoter through the FBEs. Similarly, NICD-induced promoter activity was abolished by mutating the Su(H) site (NotMT-LUC). Remarkably, Foxc2-, but not Foxc1-, induced promoter activity was also reduced by the mutation of the Su(H) site. Disruption of both the FBEs and the Su(H) site almost completely abolished the promoter activity induced by Foxc2, suggesting that Foxc2 mediates the activation of the Hey2 promoter through these functionally distinct sites.

The functional interaction between Foxc2 and the Su(H) site led us to further investigate whether Foxc and NICD in combination could synergistically induce Hey2 promoter activity in MEECs and BAECs (Figure 2B). Indeed, co-expression of Foxc2 and NICD showed the synergistic activation of the Hey2 promoter compared with expression of each molecule alone, while Foxc1 showed no synergistic effects with NICD. Surprisingly, the mutation of the Su(H) site alone greatly abolished the synergism between Foxc2 and NICD (Figure 2C). The cooperative activity of Foxc2 and NICD was completely inhibited by mutating the FBEs and the Su(H) site, whereas the combined activity remained largely intact in the mutation of the FBEs only. These findings indicate that Foxc1 and Foxc2 directly regulate the Hey2 promoter in endothelial cells and that Foxc2 functionally cooperates with Notch signaling for the induction of Hey2 promoter activity.

### Foxc2 physically interacts with Su(H)

**Figure 3:**
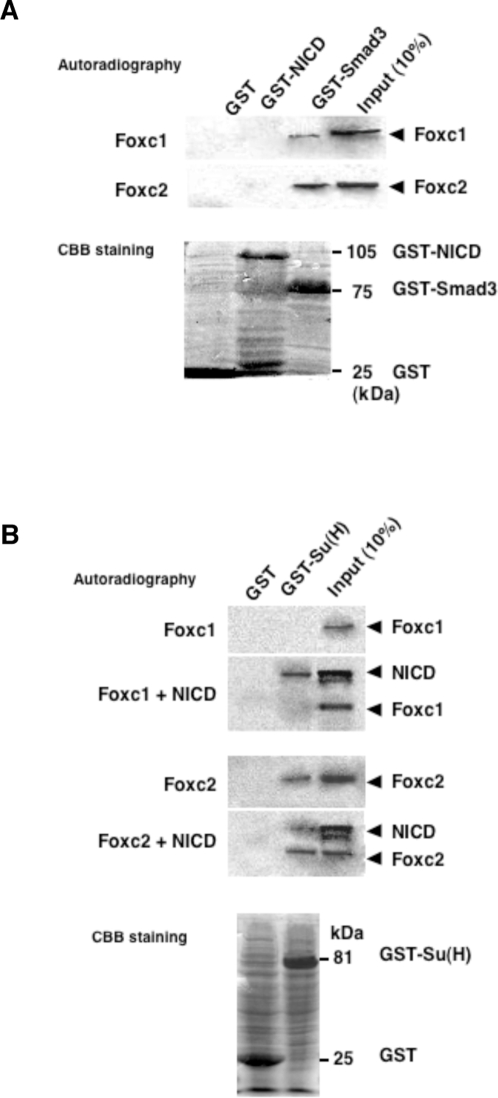
Foxc2 directly binds to Su(H). (A, B) GST-pull down assays. (A) Foxc1 and Foxc2 proteins do not physically interact with NICD. [^35^S]methionine-labeled Foxc proteins were incubated with GST-NICD or GST–Smad3 as a control and were subjected to SDS-PAGE. The gels were first stained by CBB to detect GST-fusion proteins (lower panel). Foxc proteins that bind to GST-fusion proteins were subsequently visualized by autoradiography (upper panel). (B) Foxc2 directly binds to Su(H) and forms a protein complex with Su(H) and NICD. [^35^S]methionine-labeled NICD and Foxc proteins were mixed with GST-Su(H), and GST pull-down assays were performed.

Since Foxc2 and NICD functionally interact with each other to activate the Hey2 promoter, we tested physical interactions of Foxc proteins with NICD and Su(H). We first examined whether ^35^S-labeled Foxc proteins could directly bind to GST-NICD by GST pull-down assays. Although both Foxc1 and Foxc2 bound to Smad3, as previously shown [23], neither Foxc1 nor Foxc2 physically interacted with NICD (Figure 3A). On the other hand, Foxc2, but not Foxc1, directly bound to GST-Su(H) (Figure 3B). Interestingly, when Foxc2, NICD and Su(H) were incubated together, the three proteins formed a protein complex (Figure 3B). Coupled with the results obtained from the luciferase reporter assays (Figure 2), these findings suggest that Foxc2 is directly involved in the NICD-Su(H)-mediated transcriptional activation of Hey2.

### VEGF signaling activate the Dll4 and Hey2 promoters

**Figure 4:**
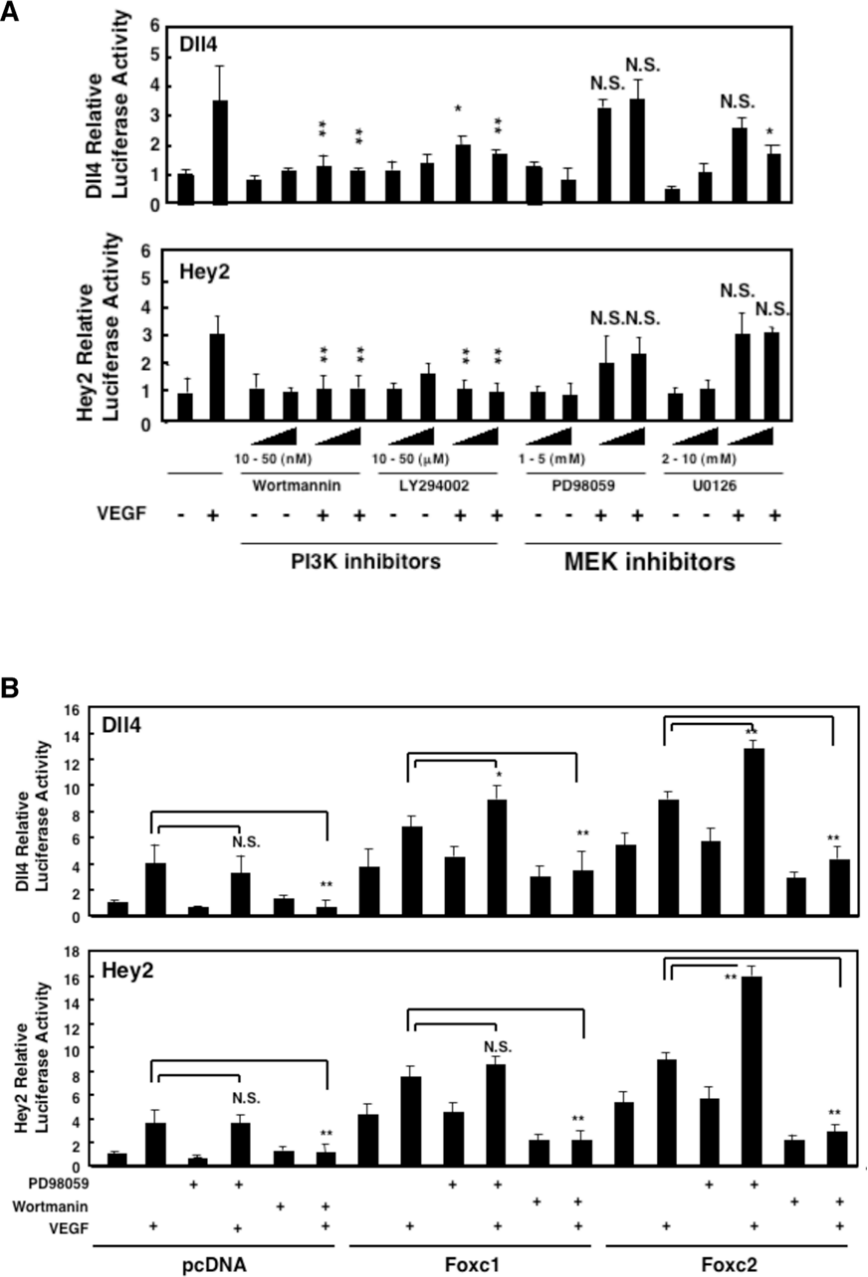
Foxc proteins interact with VEGF signaling in the activation of the Dll4 and Hey2 promoters. (A) The VEGF-induced PI3K pathway activates the Dll4 and Hey2 promoters. BAECs transfected with DLL4-LUC or Hey2-LUC at 24 h after transfection and serum starvation were treated with either PI3K inhibitors (Wortmannin and LY294002) or MEK inhibitors (PD98059 and U0126) along with VEGF (50 ng/ml) for additional 24 h. Luciferase activity was assayed at 48 h after transfection. (B) Transcriptional activity of Foxc proteins is modulated by VEGF-mediated PI3K and MEK pathways. BAECs transfected with Foxc expression vectors along with DLL4-LUC or Hey2-LUC at 24 h after transfection and serum starvation were treated with VEGF (50 ng/ml) in the presence of Wortmannin or PD98059 for additional 24 h. Luciferase activity was assayed at 48 h after transfection. Values are means+s.d. of 3 experiments in triplicates. Statistical significance was determined by Student's *t*-tests. (*p<0.05, **p<0.01 versus control cells treated with VEGF)

Although VEGF induces the expression of arterial-specific genes such as Dll4 in endothelial cells [29]–[31], VEGF-responsive elements upstream of these genes has not yet been characterized. Therefore, we analyzed the effects of VEGF and its intracellular signaling pathways on the activation of the Hey2 promoter (0.5 kb), as well as the previously reported Dll4 promoter (3.7 kb) whose activity is regulated by Foxc1 and Foxc2 via the FBE [18]. First, VEGF treatment alone significantly activated the Dll4 and Hey2 promoters in BAECs (Figure 4A), suggesting that the promoter regions used are able to respond to VEGF in endothelial cells. More importantly, PI3K inhibitors (Wortmannin and LY294002) almost completely suppressed VEGF-induced luciferase activity in BAECs, whereas treatment with MEK inhibitors (PD98059 and U0126) largely showed no effects on the responsiveness of VEGF to the Dll4 and Hey2 promoters (Figure 4A).

### Transcriptional activity of Foxc proteins is modulated by VEGF signaling

While VEGF acts upstream of Notch signaling in arterial differentiation, we have previously shown that Foxc1 and Foxc2 are expressed in both arterial and venous endothelial cells in the early mouse embryo [18]. Given that VEGF stimulation does not increase the expression of Foxc1 and Foxc2 in endothelial cells [32], [33] (Figure S1), we tested whether VEGF signaling itself could regulate the activity of Foxc proteins in the activation of the Dll4 and Hey2 promoters in BAECs (Figure 4B). First, overexpression of Foxc1 and Foxc2 along with VEGF treatment induced higher activity of the Dll4 and Hey2 promoters than Foxc expression alone. More significantly, VEGF treatment along with PI3K and MEK inhibitors revealed that blocking PI3K signaling inhibited Foxc2-mediated activation of the Dll4 and Hey2 promoters, whereas blocking ERK signaling enhanced Foxc2 action. Similarly, Dll4 induction by Foxc1 was blocked by PI3K inhibition, whereas Hey2 induction by Foxc1 was augmented by ERK inhibition. Taken together, these results indicate that VEGF signaling, specifically the PI3K component, augments Foxc-induced promoter activity of Dll4 and Hey2.

## Discussion

**Figure 5:**
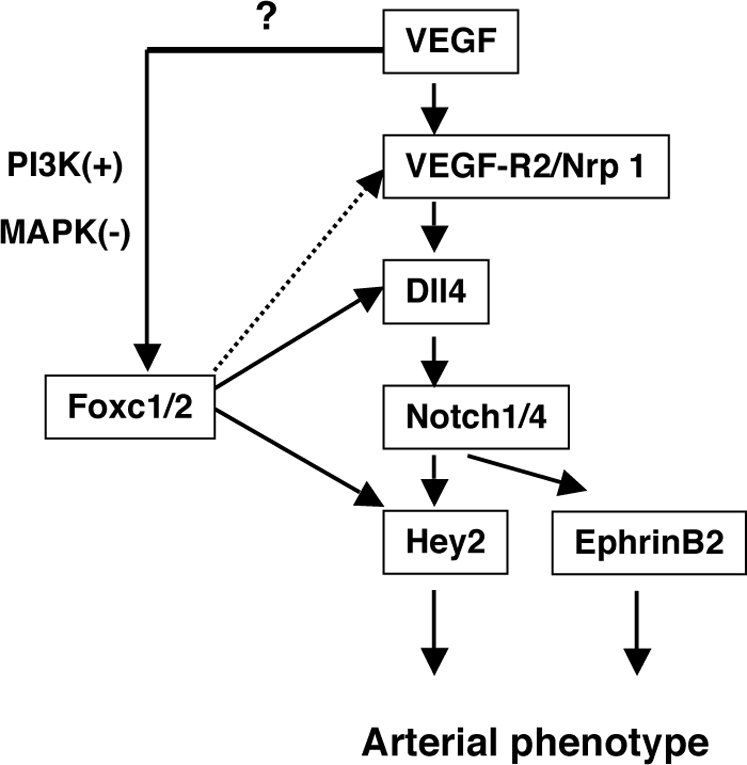
Model for the involvement of Foxc proteins in arterial gene expression program. Upon VEGF stimulation, the expression of Notch signaling genes, including the Dll4 ligand and the Notch1/4 receptors, as well as downstream targets of Notch signaling, including Hey2 and ephrinB2 [44], is induced in endothelial cells. Foxc1 and Foxc2 transcription factors directly control Dll4 and Hey2 transcription, while it remains speculative whether Foxc proteins function downstream of VEGF. Foxc2 is involved in the Su(H)-NICD transcriptional complex for the induction of Hey2 expression. Foxc proteins may also regulate expression of Nrp1, the arterial-specific co-receptor for VEGF (dashed arrow), thereby controlling the positive feedback loop of VEGF signaling.

We have previously shown that Foxc1 and Foxc2 have a dose-dependent role in arterial specification and that they directly regulate Dll4 expression. Our new findings in this paper demonstrate that Foxc1 and Foxc2 directly activate the Hey2 promoter through the FBEs and that Foxc2 cooperates with the Su(H)-NICD transcriptional complex in the induction of Hey2. Furthermore, Foxc1 and Foxc2 cooperate with the VEGF signaling pathway to activate the Dll4 and Hey2 promoters. To our knowledge, this is the first demonstration of the basic mechanisms that control arterial gene expression in concert with the VEGF and Notch signaling pathways, and such novel regulatory mechanisms mediated by Foxc proteins provide new insight into the arterial program (Figure 5).

Direct activation of the Hey2 promoter by Foxc1 and Foxc2 indicates that in addition to the induction of Dll4 expression, they reinforce an arterial phenotype in endothelial cells. Notably, by forming the transcriptional complex with Su(H) and NICD, Foxc2 acts downstream of Notch signaling to activate the Hey2 promoter. Interestingly, a recent study demonstrates the functional cooperation between Foxo1 and Notch signaling in myogenic differentiation [34], indicating interactive pathways between Fox and Notch in developmental processes. The difference between Foxc1 and Foxc2 in the binding to the Su(H)-NICD complex may be attributable to diverse C-terminal regions in the two Foxc proteins (∼30% homology), compared with their N-terminal and DNA-binding domains (56% and 97% homology, respectively). It is worth noting that compound Foxc1+/−; Foxc2−/− mutants have much more severe defects in the cardiovascular system than compound Foxc1−/−; Foxc2+/− mutants [16]–[18]. While we cannot rule out the possibility that Foxc1 is involved in non-canonical Notch signaling [35], it has also been shown that Hey2 expression is Notch-independent in some cases [36], [37].

We also show in this paper that Foxc1 and Foxc2 functionally cooperate with VEGF signaling, through the PI3K pathway, to induce Dll4 and Hey2 promoter activity. Our results are consistent with evidence that the VEGF/PI3K-mediated pathway induces Notch1 and Dll4 expression in cultured human endothelial cells [30]. In contrast, in the zebrafish embryo, the PI3K pathway suppresses arterial differentiation by blocking the ERK signaling cascade [38]. The discrepancy between these *in vitro* and *in vivo* experiments is currently unclear. While the effects of a single isoform of VEGF on arterial gene expression was tested in the *in vitro* experiments, spatiotemporal activity of multiple isoforms of VEGF in the zebrafish embryo may differently affect activation of VEGF-dependent intracellular signaling pathways in endothelial cells *in vivo*. Interestingly, it is noteworthy that MAPK activity stays much longer in the zebrafish embryo than in cultured endothelial cells. In any case, it should be emphasized that in both contexts, the two VEGF-mediated components, the PI3K and ERK pathways, appear to have opposing effects [38], [39] on arterial differentiation. One possibility to explain our *in vitro* studies is that PI3K-mediated inhibition of the ERK pathway leads to the activation of Foxc proteins. While we cannot exclude the possibility that Foxc function is, in part, independent of VEGF signaling, activity of Foxc proteins may be modulated by phosphorylation in response to VEGF. This idea is supported by evidence that human FOXC1 and FOXC2 are phosphorylated by EGF stimulation [40], [41] and that human FOXC1 activity is elaborately modulated by phosphorylation levels [40], [42]. In fact, both Foxc1 and Foxc2 have 10 potential phosphorylation sites for ERK that are conserved between human and mouse (data not shown). While there is no potential ERK1/2 phosphorylation site in the DNA binding-domains, the distribution of 10 potential sites in flanking N- and C-terminal regions are rather similar between the two proteins. It is conceivable that specific residues such as potential phosphorylation sites are commonly important for the function of Foxc1 and Foxc2. Further analysis is under way to define the amino acid residues in Foxc proteins that are phosphorylated in response to VEGF.

VEGF signaling induces the expression of Neuropilin 1 (Nrp1), as a positive-feedback loop to promote the arterial program [43]. Given evidence that Nrp1 expression is downregulated in compound Foxc1; Foxc2 homozygotes [18], we have found that Foxc2 also upregulates Nrp1 expression in endothelial cells and that there is a conserved FBE between human and mouse in the 3 kb upstream region of Nrp1 (Figure S2). It is therefore possible that Foxc transcription factors also regulate Nrp1 expression, thereby regulating the positive-feedback loop of VEGF signaling (Figure 5). In conclusion, our results demonstrate that Foxc1 and Foxc2 are important transcriptional regulators in the arterial program by interacting with VEGF and Notch signaling.

## Supporting Information

Figure S1 Expression levels of Foxc1 and Foxc2 in MEECs treated with VEGF. RNA samples were prepared after treatment with VEGF at indicated concentrations for 24 hr, and relative mRNA levels of Foxc1 and Foxc2 were measured by real-time RT-PCR. Results are presented as means+/−s.d. from triplicate experiments.
(0.02 MB PDF)

Figure S2 Foxc2 upregulates Neuropilin 1 expression in endothelial cells. MEECs were infected with recombinant adenovirus expressing Foxc2 and GFP or control adenovirus expressing GFP only (Mock). Neuropilin 1 (Nrp1) mRNA was detected by semi-quantitative RT-PCR. Gapdh was used as an internal control. (B) Identification of a conserved Foxc-binding element in the upstream region of Neuropilin 1. Human and mouse sequences in the Neuropilin 1 locus are aligned using mVISTA to identify highly conserved regions. Putative Fox-biding elements are marked by red boxes.
(0.22 MB PDF)
