# Supplementary figures and images for: Foxc Transcription Factors Directly Regulate Dll4 and Hey2 Expression by Interacting with the VEGF-Notch Signaling Pathways in Endothelial Cells
Source: PLoS One. 2008 Jun 11;3(6):e2401. doi: 10.1371/journal.pone.0002401 (PMC2398774; doi:10.1371/journal.pone.0002401)

**Figure S1**

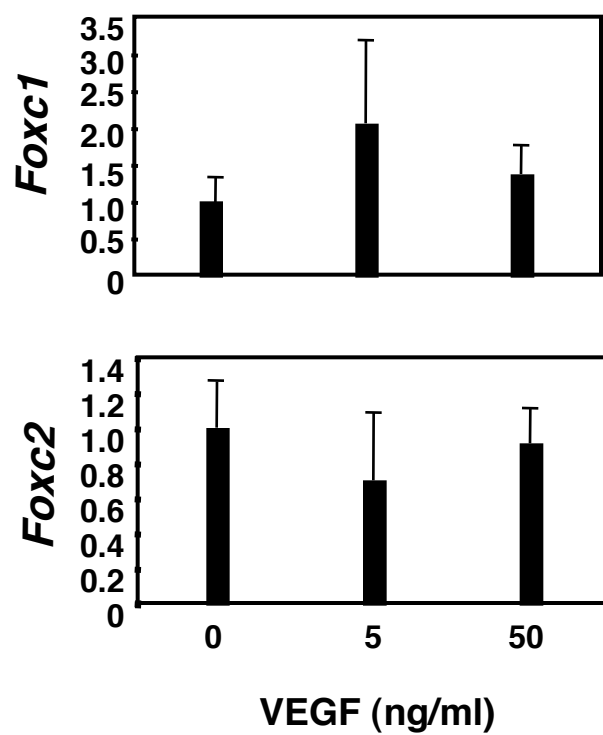

Supplement: Figure S1 — Expression levels of Foxc1 and Foxc2 in MEECs treated with VEGF. RNA samples were prepared after treatment with VEGF at indicated concentrations for 24 hr, and relative mRNA levels of Foxc1 and Foxc2 were measured by real-time RT-PCR. Results are presented as means+/−s.d. from triplicate experiments. (0.02 MB PDF) [file pone.0002401.s001.pdf]

Figure S2

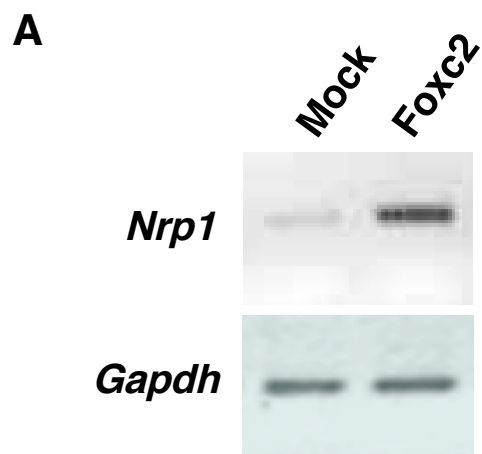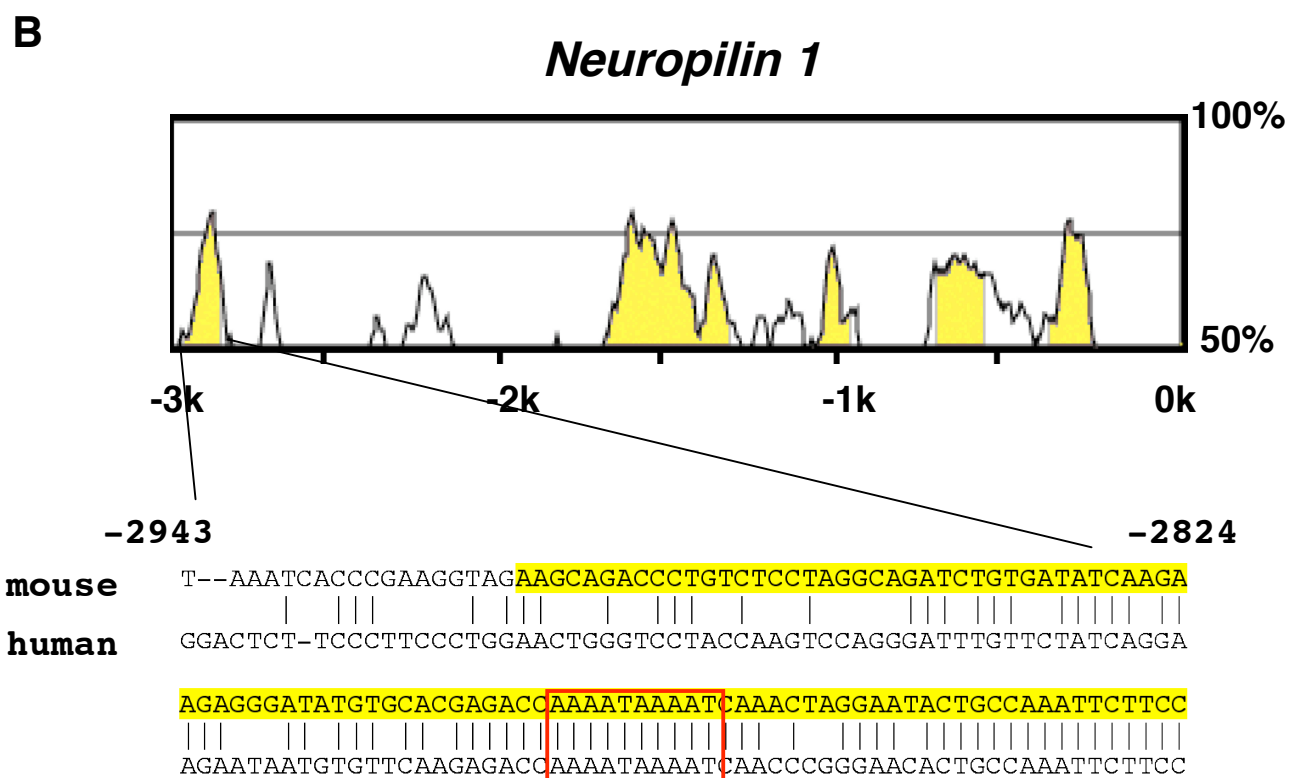

Supplement: Figure S2 — Foxc2 upregulates Neuropilin 1 expression in endothelial cells. MEECs were infected with recombinant adenovirus expressing Foxc2 and GFP or control adenovirus expressing GFP only (Mock). Neuropilin 1 (Nrp1) mRNA was detected by semi-quantitative RT-PCR. Gapdh was used as an internal control. (B) Identification of a conserved Foxc-binding element in the upstream region of Neuropilin 1. Human and mouse sequences in the Neuropilin 1 locus are aligned using mVISTA to identify highly conserved regions. Putative Fox-biding elements are marked by red boxes. (0.22 MB PDF) [file pone.0002401.s002.pdf]
